# Comparison of the Effect of High-Intensity Laser Therapy and Quadriceps Muscle Strengthening Exercises Using Biofeedback on Pain, Stiffness and Function of Patients with Knee Osteoarthritis: A Randomized Clinical Trial

**DOI:** 10.5812/aapm-143642

**Published:** 2024-12-06

**Authors:** Maryam Sadat Rahimi, Amir Masoud Jafari-Nozad, Fatemeh Jazebi

**Affiliations:** 1 Cardiovascular Diseases Research Center, Birjand University of Medical Sciences, Birjand, Iran; 2 Student Research Committee, Birjand University of Medical Sciences, Birjand, Iran

**Keywords:** Biofeedback, High Intensity Laser Therapy, Knee Osteoarthritis, Pain

## Abstract

**Objectives:**

This study aims to compare the effects of high-intensity laser therapy (HILT) and quadriceps muscle strengthening exercises using biofeedback on pain and function in patients with knee osteoarthritis (KOA).

**Methods:**

This randomized, two-group clinical trial included patients with KOA (grades II - III of the Lawrence Kellgren classification) who met the inclusion criteria. Written informed consent was obtained from participants before they were randomly allocated into one of two groups: HILT + therapeutic exercise (group A) or quadriceps muscle strengthening exercises using biofeedback + therapeutic exercise (group B). Both groups followed the same therapeutic exercise regimen during the study.

Knee pain severity was evaluated using the Visual Analogue Scale (VAS), and functional disability was assessed with the Western Ontario and McMaster Universities Osteoarthritis Index (WOMAC) questionnaire before the intervention.

For group A, HILT was performed using a BTL-6000 HIL device (wavelength 1064 nm, maximum power 12 W) following the manufacturer-recommended protocol. A pain relief program (10 W, 120 J/cm²) was administered for 120 seconds per session over ten sessions. Treatment protocol, laser positioning, and session duration were standardized. Two follow-up assessments (immediately and one-month post-intervention) were conducted to evaluate outcomes based on the VAS and WOMAC scores.

**Results:**

The study included 40 participants with KOA, divided evenly between the two groups (20 in each). The average age of the participants was 59.34 ± 6.92 years. High-intensity laser therapy group (group A): Visual analogue scale pain scores decreased significantly immediately after and one month post-intervention compared to baseline (P < 0.01). However, the VAS score one month after the intervention showed no significant difference compared to the immediate post-intervention score (P = 0.59). Biofeedback group (group B): VAS pain scores also decreased significantly both immediately after and one month post-intervention compared to baseline (P < 0.05). The difference in VAS pain reduction between the two groups was significant, with the HILT group showing greater improvement immediately after the intervention and one month later (P = 0.007).

**Conclusions:**

The study findings suggest that both quadriceps muscle strengthening exercises using biofeedback and HILT effectively reduce pain in KOA patients. However, HILT demonstrated superior efficacy compared to biofeedback exercises. These results support the use of HILT as a noninvasive therapeutic modality for KOA, particularly for patients with a higher risk of surgery due to preexisting comorbidities.

## 1. Background

Knee osteoarthritis (KOA) is an extremely debilitating condition, primarily affecting older individuals. Its main symptoms include pain (which intensifies with physical activity and weight-bearing), stiffness, and restricted range of motion (ROM) (1). This musculoskeletal disorder significantly impacts pain levels, functional ability, and overall quality of life (2). Knee osteoarthritis develops due to complex interactions between tissue damage and inflammation, with severe inflammatory responses often correlating with more intense pain and accelerated disease progression (3, 4). Prostaglandin E2 and matrix metallopeptidases are key contributors to cartilage destruction and chondrocyte loss in KOA (5).

The prevalence of KOA has risen sharply in recent years, driven by lifestyle changes, obesity, and increased life expectancy (6). Due to its progressive nature, KOA treatment typically begins with conservative approaches focused on symptom management and may eventually require advanced physical modalities or invasive surgical interventions (7). Muscle-strengthening exercises are a cornerstone of treatment plans for KOA, with numerous studies highlighting the benefits of physical therapy for these patients (5, 8, 9).

Electromyography (EMG)-biofeedback is a technique that uses equipment to provide patients with visual and auditory cues about their muscle activity. This feedback helps individuals gain better voluntary control over muscle activation or relaxation, particularly following injury (10). Biofeedback-assisted training programs are particularly valuable for improving adherence to exercise regimens and enhancing patient motivation (11). Several studies have demonstrated the effectiveness of biofeedback when combined with quadriceps strengthening exercises in KOA patients (12, 13). Laser therapy is another emerging rehabilitation modality for managing musculoskeletal disorders. Its popularity stems from its minimal side effects and versatility. The efficacy of laser therapy depends on various factors, including power output, wavelength, duration, and dosage (8). Among these modalities, high-intensity laser therapy (HILT) has gained prominence as a novel treatment for pain reduction and management of musculoskeletal conditions (2, 14). High-intensity laser therapy is known for its ability to reduce pain and inflammation while promoting the healing process (15, 16). Research has consistently shown that HILT can provide significant benefits to KOA patients, including pain relief, improved ROM, and enhanced functional performance. These outcomes are attributed to the high output power of HILT, which facilitates deep tissue penetration and delivers substantial energy in a short time (15, 17).

Most prior studies have focused on the effects of low-level laser therapy (LLLT) in the management of osteoarthritis. Given that LLLT was introduced earlier than HILT, it is unsurprising that fewer studies have investigated the HILT protocol.

## 2. Objectives

This study aimed to compare the efficacy of HILT and quadriceps muscle strengthening exercises using biofeedback in terms of pain reduction and functional improvement in patients with KOA.

## 3. Methods

### 3.1. Study Design

This study employed a randomized, two-group clinical trial design to compare the efficacy of HILT with quadriceps muscle strengthening exercises using biofeedback. The research was approved by Birjand University of Medical Sciences (BUMS) and registered with the Iranian Registry of Clinical Trials (IRCT20221115056509N1). The study protocol received ethical approval from the Ethics Committee of Birjand University (IR.BUMS.REC.1401.083).

### 3.2. Participants

The study population consisted of 40 individuals with KOA who were referred to the physical medicine and rehabilitation center at Imam Reza Hospital, Birjand, Iran, from April 2022 to January 2023. The sample size was determined based on previous similar investigations (18, 19). After receiving a detailed explanation of the intervention, all participants signed written informed consent for study participation. The diagnosis of KOA was confirmed using the criteria set by the American College of Rheumatology (ACR), and the disease stage was determined through standing knee anteroposterior (AP) weight-bearing radiographs. Patients with stage two or three KOA, as per the Lawrence Kellgren classification, were included in the study.

- Inclusion criteria: (1) Stage two or three KOA, (2) history of continuous knee pain caused by osteoarthritis for at least 6 months, (3) mechanical knee pain intensity ≥ 3 cm on the Visual Analog Scale (VAS) before the intervention, (4) Body Mass Index (BMI) ≤ 30 kg/m², (5) no use of non-steroidal anti-inflammatory drugs (NSAIDs) or steroids before or during the intervention, (6) written consent to participate in the study.

- Exclusion criteria: (1) Patients under 18 years old, (2) pregnancy, (3) unwillingness to continue the intervention, (4) irregular attendance in the treatment program, (5) use of NSAIDs or steroids, (6) intra-articular injections within the last year, (7) history of injury or surgery in the knee or other joints of the lower extremities, (8) neuromuscular disorders or malignant tumors, (9) bone implants in the lower extremities, (10) participation in other exercise therapy or physiotherapy programs during the last 6 months, Participants meeting the inclusion criteria and agreeing to the study procedures were enrolled in the trial.

### 3.3. Randomization

Participants who met the inclusion criteria were randomly assigned to either HILT + therapeutic exercise (group A) or quadriceps strengthening exercises using biofeedback + therapeutic exercise (group B) using the block randomization method. The HILT intervention consisted of 10 sessions, held every other day for 10 minutes. Group B underwent quadriceps muscle strengthening exercises using biofeedback and therapeutic exercise for 10 sessions, each lasting 30 minutes every other day.

### 3.4. Quadriceps Strength Training

The quadriceps strengthening program included three sets of isometric knee exercises (10 repetitions per set, holding for 10 seconds each) and straight leg raises held for 15 seconds, repeated 10 times daily. Participants were regularly contacted to ensure adherence to the therapy programs and to monitor for any complications. Both groups performed the same therapeutic exercises throughout the study.

### 3.5. Laser Therapy

For HILT, a BTL-6000 HIL device (wavelength 1064 nm, maximum power 12 W) was used, following the manufacturer’s recommended protocol. A pain relief program with a power of 10 W and an energy density of 120 J/cm² was administered for 120 seconds per session. All 10 sessions followed identical protocols regarding HILT positioning and treatment duration.

### 3.6. Biofeedback Settings

The single-channel MyoTrac Infiniti Continence Suite electromyographic biofeedback device (Thought Technology, Montreal, Canada) was programmed for the muscle strengthening protocol. To reduce impedance, the electrode sites on the skin were shaved and cleaned with ethyl alcohol. Electrodes were placed following the SENIAM electrode placement protocol. The active electrode was positioned 4 cm above the patella, the reference electrode 3 cm medially to the superomedial aspect, and the ground electrode on the ipsilateral leg 2 - 3 cm below the patella.

### 3.7. Outcomes

The study evaluated outcomes such as pain, stiffness, and physical functioning at three intervals: Baseline, post-intervention, and one month post-intervention. Validated questionnaires assessed pain and function before and after the trial.

#### 3.7.1. Visual Analogue Scale

The VAS is a validated index to measure pain severity. A 10 cm line scored pain from 0 (no pain) to 10 cm (worst pain). Visual Analogue Scale has been reported as a more reliable measure than other pain rating scales for KOA patients (20).

#### 3.7.2. Western Ontario and McMaster Universities Osteoarthritis Index

The Western Ontario and McMaster Universities Osteoarthritis Index (WOMAC) is a reliable and valid patient-reported instrument for assessing osteoarthritis severity (21, 22). It includes subscales for pain (5 items), stiffness (2 items), and physical functioning (17 items). Its validity and reliability in the Persian population were confirmed by Ebrahimzadeh et al. (23). The baseline characteristics of participants were examined and presented in Table 1, showing no significant differences between the two groups in pre-therapy values.

**Table 1.** Demographic Information at the Baseline

Variables	Study Groups		Total	P-Value
	Quadriceps Muscle Strengthening Exercises Using Biofeedback	HILT		
**Age**	59.91 ± 5.28	58.61 ± 8.69	59.6 ± 34.92	0.14
**BMI**	27.18 ± 2.12	26.60 ± 1.99	26.1 ± 89.03	0.3

Abbreviations: HILT, high-intensity laser therapy; BMI, Body Mass Index.

### 3.8. Statistical Analysis

Statistical analysis was performed using SPSS version 22 software (SPSS Inc., Chicago, IL, USA). Descriptive data were reported as mean ± standard deviation (SD) or number (percent). Differences in parameters between groups were analyzed using independent sample *t*-tests for continuous variables at the trial's conclusion. Changes within groups from pre- to post-intervention were assessed using paired *t*-tests. A P-value < 0.05 was considered statistically significant.

## 4. Results

A total of 40 participants with KOA completed the trial and were included in the final analyses, with 20 individuals in the HILT group and 20 in the biofeedback group. The study population consisted of 50% men (20 participants) and 50% women (20 participants), with an average age of 59.34 ± 6.92 years. Most participants had bilateral knee osteoarthritis, and diabetes was the most common comorbidity.

At baseline, there were no significant differences in the VAS pain scores between the two groups (P = 0.281). However, the VAS pain scores immediately after the intervention and one month post-intervention were significantly lower in the HILT group compared to the biofeedback group.

- HILT group (group A): The VAS pain scores immediately after and one month post-intervention were significantly reduced compared to baseline (P < 0.01). However, there was no significant difference in the VAS pain scores between the immediate post-intervention and one month post-intervention assessments (P = 0.59).

- Biofeedback group (group B): Similarly, VAS pain scores immediately after and one month post-intervention were significantly reduced compared to baseline (P < 0.05). The reduction in VAS pain scores between the two follow-up durations (immediate and one month post-intervention) was also significant (P = 0.007).

WOMAC Scores:

- HILT group (group A): WOMAC scores were significantly lower immediately after and one month post-intervention compared to baseline (P = 0.009 and P = 0.008, respectively). Additionally, the change in WOMAC scores between the immediate post-intervention and one month post-intervention assessments was significant (P = 0.045).

- Biofeedback group (group B): The WOMAC scores immediately after and one month post-intervention were also significantly reduced compared to baseline (P = 0.039 and P = 0.002, respectively). Furthermore, WOMAC scores one month post-intervention were significantly lower compared to scores immediately after the intervention (P = 0.015).

Despite these intra-group improvements, there were no significant differences in WOMAC scores between the HILT and biofeedback groups at baseline or at the two follow-up points (P > 0.05).

Tables 2 and 3 provide detailed comparisons of VAS and WOMAC scores across the groups and follow-up intervals.

**Table 2.** Differences Between the Two Groups in Terms of Visual Analogue Scale and Western Ontario and McMaster Universities Osteoarthritis Index Scores

Variables	Study Groups		P-Value
	HILT	Biofeedback	
**VAS score before the intervention**	5.45 ± 1.66	5.80 ± 1.19	0.281
**VAS score immediately after the intervention**	4.00 ± 1.71	5.25 ± 1.02	0.006
**VAS score 1 month after the intervention**	3.90 ± 1.48	4.80 ± 0.95	0.018
**WOMAC score before the intervention**	51.40 ± 16.26	51.35 ± 16.20	0.978
**WOMAC score immediately after the intervention**	48.40 ± 14.71	49.95 ± 15.19	0.607
**WOMAC score 1 month after the intervention**	44.55 ± 14.67	48.50 ± 15.47	0.357

Abbreviations: VAS, Visual Analogue Scale; WOMAC, Western Ontario and McMaster Universities Osteoarthritis Index; HILT, high-intensity laser therapy.

**Table 3.** Changes in Measured Variables in Each Group in Follow up Periods

Variables	P-Value	
	HILT	Biofeedback
**VAS score before the intervention and immediately after the intervention**	0.002	0.012
**VAS score before the intervention and 1 month after the intervention**	0.002	0.001
**VAS score immediately after the intervention and 1 month after the intervention**	0.59	0.007
**WOMAC score before the intervention and immediately after the intervention**	0.009	0.039
**WOMAC score before the intervention and 1 month after the intervention**	0.008	0.002
**WOMAC score immediately after the intervention and 1 month after the intervention**	0.045	0.015

Abbreviations: VAS, Visual Analogue Scale; WOMAC, Western Ontario and McMaster Universities Osteoarthritis Index; HILT, high-intensity laser therapy.

## 5. Discussion

There is growing interest in using safe and efficient physical modalities for treating musculoskeletal disorders. High-intensity laser therapy is a novel approach applied in various musculoskeletal conditions (19), though its use in KOA remains underexplored. High-intensity laser therapy has shown promise in reducing pain and inflammation while promoting healing (2, 15). This study aimed to evaluate and compare the effects of HILT and quadriceps muscle strengthening exercises using biofeedback on pain and function in patients with KOA. Our findings demonstrated that HILT significantly reduced both the VAS pain score and the WOMAC score compared to baseline measurements. Interestingly, HILT outperformed quadriceps strengthening exercises using biofeedback in reducing the VAS pain score in the short term and was more effective in maintaining this reduction at the one-month follow-up.

A recent systematic review highlighted that HILT may effectively reduce pain and improve function in KOA patients (8). Angelova and Ilieva concluded that HILT could be considered a treatment of choice for pain reduction and functional improvement in KOA (24). The analgesic effects of HILT are likely linked to its ability to modulate the release of proteins like bradykinin and histamine from injured tissues and elevate the pain threshold by increasing the release of substance P (25, 26). High-intensity laser therapy also enhances blood flow, improves lymphatic drainage, and reduces swelling (17, 27).

Physical therapy is well-documented for its anti-inflammatory effects, reduction of pain sensitivity, enhancement of physical functioning, and potential to improve cartilage morphology (28, 29). Combining rehabilitation exercises with laser therapy is an established procedure for KOA treatment. However, the exact efficacy of these combined methods remains under investigation. Ahmad et al. reported the effectiveness of both LLLT and HILT when combined with rehabilitation exercise in reducing KOA symptoms, with HILT showing greater improvements in pain, stiffness, and function (30). Akaltun et al. similarly noted significant improvements in VAS and WOMAC scores in KOA patients treated with HILT over six weeks (26). Additionally, Alayat et al. observed superior reductions in VAS and WOMAC scores in patients undergoing glycosaminoglycan/chondroitin sulfate treatment combined with HILT and exercise therapy compared to other groups (31).

Recent research suggests that HILT is superior to other therapy modalities. Mostafa et al. reported that HILT was more effective than shock wave therapy in reducing pain, decreasing disability, and enhancing physical function in KOA patients (32). Another study found that HILT outperformed conventional therapy modalities for pain relief and functional improvement in KOA (2). Nazari et al. revealed that HILT was significantly more effective than traditional physical and exercise therapy in reducing VAS and WOMAC scores after a 12-week follow-up. They recommended HILT combined with exercise therapy as a favorable treatment approach for KOA patients (33).

Our results support the conclusion that both quadriceps muscle strengthening exercises and HILT are effective in reducing pain and improving function in KOA patients, with HILT emerging as the more effective therapeutic modality.

### 5.1. Study Limitations and Future Directions

This study had limitations, including a small sample size and the absence of a control group. Future research with larger populations is necessary to investigate the effects of HILT across varying doses and durations on KOA patients. Further exploration may solidify HILT's role as a cornerstone in KOA management.

### 5.2. Conclusions

Our findings revealed that although both quadriceps muscle strengthening exercises using biofeedback and laser therapy are effective in reducing pain in KOA patients, HILT—especially when combined with an appropriate exercise regimen—is the more effective therapeutic modality in the short term. Furthermore, its pain-reducing effects were significantly greater in the one-month follow-up. These results emphasize the potential of HILT as a noninvasive therapeutic option for KOA, particularly for patients with an elevated risk of surgery due to comorbidities.

## Data Availability

The datasets used and analyzed during the current study are available from the corresponding author on reasonable request.

## Clinical Trial Registration Code:

This work was approved by the Birjand University of Medical Sciences (BUMS) and registered at the Iranian Registry of Clinical Trial (IRCT20221115056509N1).
